# Resilience as Safety Culture in German Emergency Medical Services: Examining Irritation and Burnout

**DOI:** 10.3390/healthcare12181860

**Published:** 2024-09-15

**Authors:** Beatrice Thielmann, Malwine Ifferth, Irina Böckelmann

**Affiliations:** Institute of Occupational Medicine, Faculty of Medicine, Otto von Guericke University Magdeburg, Leipziger Str. 44, 39120 Magdeburg, Germany; m.ifferth@t-online.de (M.I.); irina.boeckelmann@med.ovgu.de (I.B.)

**Keywords:** stress management, health promotion, prevention, paramedic, job satisfaction, teamwork, support systems, training programs, safety culture, mental well-being

## Abstract

Background/Objectives: The stress levels in emergency services are enormous. The resulting stress can range from psychological irritation to burnout. This study examines the importance of resilience in the German EMS and its significance for the risk of irritation and burnout among EMS personnel. Methods: A quantitative cross-sectional online survey was conducted among 285 EMS personnel in Germany. Resilience was measured by the RS-13 Scale, irritation by the Irritation Scale (IS), and burnout by the Maslach Burnout Inventory (MBI). Sociodemographic and job-related data were also collected. A classification into resilient groups was used to compare stress levels. Results: More than one-third (39%) of the participants had a low level of resilience. EMS personnel with high levels of resilience had significantly lower scores on the cognitive and emotional irritation dimensions, as well as on the burnout dimensions of emotional exhaustion and cynicism. Conclusions: Resilience plays an important role in the safety culture of emergency services. The results support the hypothesis that high levels of resilience lead to less stress and help people cope better with stress. Almost two-fifths of the participants had lower resilience, underscoring the need for safe communication and targeted measures to strengthen resilience. Regular training, a supportive work environment, and promoting team cohesion and social support can improve emergency responders’ mental health and job performance. Future research should develop specific intervention strategies and evaluate their effectiveness to ensure the long-term health of emergency responders and improve the quality of emergency care.

## 1. Introduction

Rescue services play an indispensable role in providing healthcare to the population and face numerous daily challenges associated with considerable physical and mental stress [1]. As an indispensable, system-relevant component of the healthcare system, the ambulance service ensures immediate medical care and makes a significant contribution to public safety by performing a variety of tasks that go far beyond the mere emergency care of patients. In addition to operations in classic emergency scenarios, such as heart attacks, strokes, and trauma, the ambulance service is integral to disaster control and emergency plans for major incidents. In the event of natural disasters, terrorist attacks, or major accidents, emergency services coordinate on-site medical care, triage injured individuals, and transport them to suitable hospitals as quickly as possible. Another important contribution to public safety is the rescue service’s commitment to prevention and health education. Emergency services help inform the public about life-saving measures and increase general health literacy by training the public in first aid or blood donation campaigns. Cooperating with fire departments or police forces in emergencies, major damage situations, and disaster situations is also a competency of the rescue service, i.e., the rescue service works intersectorally [2]. Such events require well-coordinated cooperation between authorities and organizations with security tasks to overcome the complex challenges of the situation that arises.

The importance of a robust safety culture in companies, defined as the totality of attitudes, views, and behaviors of all employees with respect to safety, cannot be overstated, especially in the context of the emergency services organization. Since many aspects of work in emergency services, as outlined, are beyond the control of those involved, it is all the more important to be well prepared internally. The challenges of today’s world of (volatility, uncertainty, complexity, and ambiguity (VUCA) and fragility, anxiety, nonlinearity, and incomprehensibility (BANI) require special attention and adaptability from employees and companies. These dynamic and often unpredictable conditions significantly increase the physical and psychological strain on emergency services. In the following, the challenges in the VUCA and BANI world are discussed in detail, including how these factors can affect daily work in emergency services and what strategies are needed to cope with these stresses.

### 1.1. Stress in Emergency Services: Challenges in the VUCA and BANI World

The emergency services are exposed to various physical, psychological, and organizational stresses daily [1,3,4,5,6]. These stresses result not only from the immediate work at the place of deployment but also from the constantly changing and uncertain working conditions, which are characterized by the terms VUCA (volatility, uncertainty, complexity, ambiguity) [6,7] and BANI (brittle, anxious, nonlinear, incomprehensible).

VUCA applied to the emergency services:

- Volatility: The working environment in emergency services is characterized by rapid and unpredictable constant changes. Incidents can occur at any time and without warning, and the nature of emergencies varies greatly, from traffic accidents to medical emergencies such as heart attacks or strokes. Work requirements are becoming more diverse and dynamic, requiring a high degree of flexibility on the part of the emergency services. The operation is a dynamic process that can change quickly, so the initial working diagnosis does not have to be final.
- Uncertainty: Rescue workers are often confronted with incomplete or contradictory information, so treatment can be uncertain. Decisions must be made quickly, often without the opportunity to collect and analyze all relevant data. This uncertainty can lead to psychological stress as the consequences of decisions can be immediate and severe.
- Complexity: Rescue service operations are often complex and require interaction between different specialist areas and players. Coordination between rescue service personnel and, for example, control centers, fire departments, police forces, and hospitals can be difficult, especially in supraregional operations. This complexity requires technical knowledge, organizational skills, and teamwork.
- Ambiguity: Emergency responders encounter situations that are often ambiguous and unpredictable. Patient symptoms can indicate various conditions, and the best course of action is not always clear. This ambiguity can lead to decision-making pressure and mental stress as responders constantly have to weigh which action is right. One treatment decision can have immediate consequences.

VUCA makes it clear that, depending on the constellation of conditions on site, the operation can have bland or serious effects on the operation’s success, including on the health of the rescue workers.

Although the VUCA world already appears problematic and challenging, more recent concepts such as BANI indicate that the world of work can also be unstable and even chaotic [8]. The acronym BANI stands for brittle, anxious, nonlinear, and incomprehensible. The BANI concept fits well with the rescue service setting. Even if the current situation appears stable, it is still fragile and porous. This fragility can be dangerous as challenges such as a shortage of skilled workers and demographic change can threaten established structures.

The transfer of BANI to the rescue service can mean the following:

- Brittle: Emergency service infrastructure and systems can be brittle, meaning that they can easily break down under pressure. Overstretched emergency departments and inadequate equipment or staff shortages can affect the ability to respond effectively. This increases the strain on emergency personnel, who often have to work in suboptimal conditions.
- Anxiety: Constant confrontation with life-threatening situations and responsibility for other people’s lives can lead to anxiety in rescue workers. The fear of making mistakes or unforeseen events can strain their mental health.
- Nonlinear: Processes and developments in emergency services are often nonlinear. Small errors or delays can lead to major consequences, making planning and control difficult. This required the emergency services to be highly adaptable and able to react quickly to unexpected developments.
- Incomprehensible: Some events and situations in emergency services are incomprehensible and difficult to process. Traumatic experiences, such as the death of patients or being confronted with serious injuries, can have long-term psychological consequences and increase strain on emergency personnel.

The multilayered stresses in rescue services require a comprehensive understanding and targeted measures to protect the health and well-being of rescue workers. Theoretical models and concepts are essential for understanding and explaining how to address stressors and the resulting strain. They help identify and analyze various stressors, how they affect individuals, and which resources and coping strategies can be used to reduce stress. Some studies have shown that work-related stress represents a significant financial burden for societies worldwide [9,10]. The estimated costs range from EUR 54 million to EUR 280 billion depending on the country. The results indicate that the loss of productivity due to absenteeism and presenteeism is economically far more severe than the direct medical costs [9].

### 1.2. Theoretical Models to Explain Stress, Irritation, and Burnout

As developed by Mohr et al. (2005), the concept of irritation describes a form of psychological stress that encompasses both cognitive and emotional aspects [11]. Irritation is understood as a state of inner restlessness and dissatisfaction that is triggered by disturbing or stressful events. It is characterized by an ongoing mental preoccupation (rumination) with these stressful work events as well as feelings of irritation and frustration. Irritation can be seen as an early warning signal for deeper stress problems as it is a state of increased stress but does not yet indicate illness. Mohr et al. emphasized that irritation reflects not only the immediate reaction to stressors but also the inability to mentally distance oneself from them. This can lead to a downward spiral in which those affected increasingly fall into negative thought loops and have difficulty recovering [12]. In the long term, this can increase the risk of more serious health problems, such as burnout, anxiety, depression, cardiovascular disease, and neuroendocrine disorders, due to a persistent perception of stress [12,13,14,15].

The model “Allostasis and Allostatic Load” by McEwen (1998) describes how the body reacts to stress through allostatic processes to maintain homeostasis [13]. Allostasis is the adaptation through physiological changes, such as releasing stress hormones (e.g., cortisol). The allostatic load refers to the cumulative load caused by repeated or chronic stress reactions, which can lead to long-term health problems. A high allostatic load is associated with numerous physical and mental health problems, including cardiovascular disease and depression [16,17].

The job demands–resources model (JD-R) by Demerouti et al. (2001) distinguishes between job demands and resources [18,19]. Job demands include physical, psychological, social, or organizational aspects of work that require continuous effort and are, therefore, associated with physical and psychological costs. Work resources, on the other hand, are elements of work that support the achievement of work goals, reduce stress, and promote personal growth. An imbalance in which work demands predominate leads to stress and potentially burnout in the long term [18,19,20].

The transactional stress model of Lazarus and Folkman (1985) views stress as an interaction between the individual and the environment [21]. Stress arises when a person evaluates a situation as threatening or overwhelming and believes they do not have sufficient resources to cope with it. The model distinguishes between primary and secondary appraisal: in primary appraisal, a decision is made as to whether an event is irrelevant, positive, or stress-inducing. The secondary evaluation assesses whether the available coping skills and resources are sufficient to address the event. This assessment process is dynamic and continuous, which means that stress is not only determined by the objective characteristics of a situation but also significantly influenced by subjective perceptions and available coping strategies. In the long term, stress can lead to depression, among other effects [22]. A reduced, but not entirely absent, level of empathy can be helpful during an operation to provide emotional shielding. At the same time, actively engaging with one’s own emotions and processing critical situations after the operation appears to be an adaptive way to protect against stress [23].

The stress-strain concept of Rohmert and Rutenfranz (1975) is a fundamental model in occupational science for understanding and evaluating the effects of working conditions on the human organism [24]. A person’s ability to cope with the demands of workplace stress depends on their physical and psychological resources. This means that a certain level of stress can lead to different levels of strain in different people. In prevention and health promotion, in particular, this model stands for measures to strengthen individual performance and promote resilience to stress.

### 1.3. Consequences of a Permanent, Uncompensated Stress Load

As seen from the models presented above, stress is a key factor that can lead to a variety of physical and mental health problems. A chronic imbalance between work demands and available resources, as described in the job demands–resources model, or a persistently high allostatic load can lead to serious health problems in the long term. One of these consequences is burnout, a state of deep emotional, physical, and mental exhaustion caused by prolonged stress and overload in the workplace [25,26].

This condition is often caused by chronic overwork, a lack of control over the work situation, and a lack of support. People suffering from burnout often feel discouraged, lack energy, and are unable to do their work efficiently. In the 10th International Classification of Diseases (ICD-10), burnout is classified under the code Z73.0 [27]. Burnout is described as a “problem related to difficulties in coping with life”. Burnout is seen as a factor influencing the state of health rather than a disease in its own right. In the 11th revision of the International Classification of Diseases (ICD-11) published by the World Health Organization (WHO) (not yet recognized for billing diagnoses in Germany), burnout is defined more specifically and is given its own classification under the code QD85. The ICD-11 defines burnout as a syndrome resulting from chronic stress at work that is not successfully managed [28]. It comprises three dimensions: (1) feelings of loss of energy or exhaustion, (2) increased mental detachment or cynicism in relation to one’s work and (3) reduced job performance. The ICD-11 emphasizes that burnout is specific to work-related stress and cannot be transferred to other areas of life.

### 1.4. Resilient Emergency Services as a Safety Culture and Counterpart to Stress

A robust safety culture in emergency services is essential to protect both employees and patients [29]. Safety culture refers to the shared values, beliefs, and behaviors within an organization that shape safety awareness and practices.

A central aspect of this safety culture is the promotion of resilience. As part of a safety culture, resilience helps emergency services cope better with stressful and traumatic events and emerge more strongly from them. Resilience refers to the ability of individuals, teams, and organizations to recover from stress, adversity, or traumatic events and emerge more strongly from them [30]. It comprises the internal and external resources that make it possible to adapt to changing conditions and overcome challenges. Resilience is particularly important in emergency services where rescue workers are regularly exposed to extreme physical and psychological stress.

There are different aspects of resilience. Psychological resilience refers to a person’s inner mental and emotional ability to cope with stress and setbacks. Psychological resilience can be strengthened through positive self-perception, optimism, and the ability to learn from experience [31]. Social resilience emphasizes the importance of social networks and support systems. Close relationships with family, friends, and colleagues can be a buffer against stress and can promote mental health [31]. At an organizational level, resilience refers to the ability of an organization to survive crises and continue to function effectively. This includes adaptability, flexibility, and contingency plans and protocols [32].

Aaron Antonovsky’s concept of salutogenesis poses the question of what keeps people healthy, in contrast to pathogenesis, which focuses on the causes of illnesses [33]. The term sense of coherence also returns to this concept. A central component of salutogenesis is the sense of coherence (SoC), which is made up of three components: (1) comprehensibility, i.e., events are meaningful and consistent; (2) manageability, in the sense of confidence that sufficient resources are available to meet demands; and (3) meaningfulness, in which efforts to solve challenges are worthwhile [33,34].

A strong SoC promotes resilience by helping people view stressors as manageable and meaningful [33]. Resilient people are optimistic; they see every experience as educational, focus on their individual strengths and abilities, accept constructive feedback, maintain close relationships, have strong social skills, and have a good understanding of their emotions [35]. This contributes to mental health, well-being, satisfaction, and performance and is particularly relevant for emergency workers who are confronted with demanding and often traumatic situations on a daily basis [36,37].

### 1.5. Derivation of the Research Question: Resilience and Its Effects on Irritation and Burnout in the German Rescue Service

While previous studies, such as Bartone et al. (2022), have highlighted the importance of hardiness—a component of resilience—in reducing stress and burnout among healthcare and emergency service workers [38,39], the present study provides an additional perspective by examining specific dimensions of resilience as experienced in the German Emergency Medical Services (EMS). This work complements existing knowledge by analyzing the interactions between resilience, irritability, and burnout in a cultural and job-specific context. This nuance is crucial for developing targeted strategies to promote mental health and resilience within EMS.

The previous presentation highlighted the key importance of resilience in the safety culture of emergency services. In particular, in the context of the high levels of mental and physical stress to which rescue workers are exposed, the promotion of resilience is clearly of central importance. This survey should help develop a deeper understanding of the complex interactions among resilience, stress, and personal and professional characteristics. The data could provide important information for developing targeted intervention measures that help sustainably improve rescue workers’ mental health and well-being. The data on resilience or resilience levels and the outcomes of irritation and burnout in German emergency services are minimal.

In view of these findings, this study focuses on the role of resilient groups in German emergency services and their role in the consequences of stress, such as irritation and burnout. The central research question is as follows: How does the resilience level of rescue workers influence the outcome of stress in the form of irritation and burnout? A hypothesis is proposed:

**H1.**  *A higher level of resilience is correlated with lower stress, manifested in reduced cognitive and emotional irritation and a lower risk of burnout.*

Extended research question: Several sociodemographic factors (e.g., age, children, marital status) and job-related factors (e.g., work experience, location of assignments) as covariates have a strong effect on stress.

## 2. Materials and Methods

### 2.1. Study Design

As part of a quantitative cross-sectional study, German rescue service personnel were surveyed online from April to June 2023. The rescue service personnel also included emergency physicians. Participants were recruited via private contacts (two of the authors are/were active rescue service employees), social media (Facebook, LinkedIn, Instagram, Telegram), email distribution lists of universities, colleges and aid organizations, and flyers in regional rescue stations and hospitals. The study was also published in the German trade journal “Rettungsdienst” to reach other rescue service personnel throughout Germany. The questionnaire was collected via the website https://www.soscisurvey.de/ (SoSci, SoSci Survey GmbH, Munich, Germany). The study complies with the conditions of the Declaration of Helsinki (“Ethical Principles for Medical Research Involving Human Subjects”), and a positive vote from the ethics committee of Otto von Guericke University (No. 24/23) is available. Participation in the online survey was voluntary. Consent was assumed upon completion of the questionnaires.

### 2.2. Sample

Only complete datasets were included in the evaluation. Data from 285 rescue service employees were therefore analyzed. The average age of the total sample was 37.6 ± 10.4 years. A total of 72.6% of the participants were male, 26.7% were female and 0.7% were diverse.

The following inclusion criteria were defined: ambulance personnel, including paramedics, paramedic assistants, emergency paramedics, and emergency physicians, who perform this as their main profession. The age of the participants was defined as being between 18 and 67 years. People who carried out their work as a secondary occupation were excluded.

Given that there are different training occupations in the rescue service industry in Germany, a brief description of the qualifications is provided:

- RettSan (German, paramedic): Training of 520 h in 3 months (not a certified profession). As a rule, they assist the emergency paramedic.
- RettAss (German, Rettungsassistent): Two-year vocational training. This profession is no longer trained in Germany. However, valid job titles remain.
- NotSan (German, Notfallsanitäter): Three-year vocational training. It is the most common nonmedical profession in Germany.
- Emergency physicians.

All participants had to fill out a questionnaire containing the following mostly standardized questionnaires:

- Questionnaire on sociodemographic and work-related data;
- Resilience Scale (RS-13) [40];
- Irritation Scale (IS) [11];
- Maslach Burnout Inventory-General Survey (MBI-GS) [25,41] in the German version, according to Büssing and Perrar (1992) [42].

### 2.3. Methodology

#### 2.3.1. Questionnaire on Sociodemographic and Work-Related Data

This questionnaire was developed in-house. General information on sociodemographic data included age, gender, marital status or living in a partnership, and the presence of children or relatives to be cared for. Occupational data included questions on professional training and work in the rescue service (qualification), years of professional experience, area of operation (metropolis, large city, small town, rural rescue), weekly working hours, shift system, frequency of deployment per shift, and whether the person held a managerial position.

#### 2.3.2. Resilience Scale (RS-13)

Resilience refers to the ability to successfully cope with stress and difficult life situations and to emerge stronger from them. The original scale (RS-25) is adopted from Wagnild and Young (1993) [43]. The German version of the resilience scale by Wagnild and Young (1993) was evaluated in a large German population sample [44]. The revised version of the Resilience Scale RS-13 according to Leppert et al. (2008) used here is a psychological instrument that was developed to measure resilience, i.e., the psychological resilience of individuals with the subscales (personal) competence and acceptance [40]. Competence refers to confidence in one’s ability and the perception of one’s effectiveness in various areas of life. A typical example of a statement is “I have enough energy to do everything I need to do”. Acceptance refers to a person’s ability to accept themselves, their circumstances, and the inevitability of change and challenges. A selected example question is “I take things as they come”.

A total of 13 items and a seven-point Likert scale ranging from (1) “I disagree” to (7) “I completely agree” are available. The evaluation is carried out by summing the scores for all 13 items [40]:

- Points: 13–66—low resilience. Low-resilience group, resilience group I.
- Points: 67–72—medium resilience. Moderate-resilience group, resilience group II.
- Points: 73–91—high resilience. High-resilience group, resilience group III.

This instrument was used to allow comparisons with other studies by the other research groups [45,46,47,48]. A shortened RS-13 has comparable reliability, construct, and convergent validity and measures resilience well [49]. The questionnaire has been validated in representative samples [40]. The short version of the RS-13 can identify individuals who are more stressed than others [48].

The processing time is approximately 10 min. It has good internal consistency, with Cronbach’s α > 0.85. The participants in the survey were divided into three groups with low, moderate, and high resilience, and the following stress parameters were compared.

#### 2.3.3. Irritation Scale (IS)

Irritation is a state of mental impairment located between mental exhaustion and mental illness [11]. The main difference between mental fatigue and irritation lies in reactions to rest periods. Mental fatigue usually disappears after sufficient rest (e.g., rest on the weekend), whereas irritation often persists. Chronic irritability is not a mental illness but can indicate possible psychological problems. The irritation questionnaire consists of 8 items and uses a seven-point response scale ranging from (1) “strongly disagree” to (7) “strongly agree”. The instrument distinguishes between two main scales: “Cognitive irritation” (sample question “I find it difficult to switch off after work”) and “Emotional irritation” (sample question “I am easily annoyed”).

The “cognitive irritation” subscale is characterized by constant brooding about work problems. People with high scores on this subscale often think intensively about their work problems outside of working hours. They believe that these recurring thoughts help them achieve their goals. However, this ruminating is counterproductive as the constant preoccupation with existing problems hinders the ability to effectively accomplish new tasks. The “Emotional irritation” subscale measures tendencies toward verbal aggression and irritability, manifesting in grumpy reactions or frustrations. This form of irritation includes emotional reactions to work-related stressors that make it difficult to achieve goals. Emotional irritation is seen as a defensive reaction to obstacles that hinder progress. The questionnaire took approximately 4 min to complete.

The evaluation is initially carried out by adding the scores of the responses. Cognitive irritation has 3 items, and emotional irritation has 5 items. Therefore, the raw score points are between 3 and 21 points and between 5 and 35 points. The individual components are added together for the overall irritation index, resulting in scores between 8 and 56 points. Mohr et al. (2007) recommended the presentation of standardized norm values, which were recently adapted and validated by Gralla et al. (2023) via a representative German sample [50].

The IS shows an internal consistency of Cronbach’s α between 0.80 and 0.90 for emotional cognition and between 0.75 and 0.91 for cognitive irritation and the overall index.

#### 2.3.4. Maslach Burnout Inventory

Burnout is a psychological syndrome that results from chronic work stress and is characterized by three main dimensions:

- Emotional exhaustion refers to feeling overwhelmed and burnt out due to work demands. Those affected feel emotionally drained and that their emotional and physical energy is depleted. An example of a statement is “I feel emotionally empty at work”.
- Cynicism/depersonalization describes a distanced and cynical attitude toward the recipients of one’s own work, such as customers or patients. People who suffer from depersonalization tend to treat their work tasks with indifference or negativity. A typical response is “I just want to get my work done and otherwise be left alone”.
- Personnel accomplishment measures feelings of inadequacy and a lack of professional achievement. Affected people feel that they cannot do their work effectively and experience a decline in their competence and sense of achievement in their job. The following statement is an example: “I can effectively solve the problems that arise in my work”.

The Maslach Burnout Inventory (MBI) is a widely used and recognized instrument for measuring burnout [26]. The German version of the Maslach Burnout Inventory-General Survey (MBI-GS) used here comprises 16 items [41]. The last four weeks should be taken into account when answering the questions. The answers are rated on a seven-point frequency scale from “0 never” to “6 daily”. It took approximately 10 min to answer the questions. According to Maslach and Jackson, the evaluation is carried out by adding the scores of the questions assigned to the dimensions and then calculating the mean value for each of the three MBI dimensions (emotional exhaustion, cynicism, and performance). For the three-dimensional construct, the values of the three dimensions can be divided into low, average, and high values (see Table 1).

According to Kalimo et al. (2003), the assessment assesses the risk of burnout [51]. For this purpose, the MBI dimensions are weighted, or the performance is recoded beforehand into reduced performance. A higher score is associated with a greater frequency of symptoms and, therefore, an increased risk of burnout, as follows:

- Points: 0–1.49—no burnout or symptoms a few times a year;
- Points: 1.5–3.49—some burnout symptoms and symptoms once a month;
- Points: 3.5–6—burnout risk with symptoms several times a week or daily.

The MBI shows an internal consistency of Cronbach’s α between 0.79 and 0.84 for the MBI dimensions.

### 2.4. Statistical Analyses

SPSS (version 28.0.0.0, IBM, New York, NY, USA) was used for the statistical analyses. The descriptive statistics included the mean, standard deviation, median, minimum, maximum, and 95% confidence interval. The evaluation of the data distributions for normality revealed non-normal distributions. The significance level α was set at 5%. The chi-square test was used to compare the distributions of the categorical variables in the resilient groups. The Kruskal–Wallis test and, if significant, the subsequent pairwise comparison with the Bonferroni correction, were used to compare the variables between the resilient groups. Finally, a Spearman correlation analysis was carried out for the correlation questions. Following Cohen 1988, Spearman’s rho was classified as weak correlation ρ = 0.10, moderate correlation ρ = 0.30, and strong correlation ρ = 0.50 [52]. Finally, an assessment was made for possible predictors of resilience as part of a multivariate test with a test for between-subjects effects. The interpretation was carried out according to the following scheme: η^2^ < 0.06 (mild effect), η^2^ = 0.06 to 0.14 (moderate effect), and η^2^ > 0.14 (high effect) [52]. First, a GLM analysis with bootstrap specifications of the job-related data was conducted, taking into account factors such as age, work experience, and resilience rating. The dependent variables included cognitive, emotional, and total irritation as well as emotional exhaustion, cynicism, and performance. Second, the sociodemographic data were analyzed to examine the effects of age, work experience, marital status, children, qualifications, and managerial function on psychological stress in the three resilience groups.

## 3. Results

### 3.1. Presentation of the Sociodemographic and Occupational Data of the Overall Sample and Resilient Groups

#### 3.1.1. Demographic Characteristics

This study examined three resilient groups in German emergency services: high-resilience (High) (n = 117), moderate-resilience (Moderate) (n = 58), and low resilience (Low) (n = 110) groups. Statistical analysis revealed significant differences between these groups in several demographic and occupational characteristics. The high-resilience group was older on average (40.4 ± 10.5 years) than the moderate (34.4 ± 8.6 years) and low-resilience groups (36.5 ± 10.5 years) (*p* < 0.001). Male participants dominated in all groups, with 44.4% of the men being highly resilient, 21.3% moderately resilient, and 34.3% having low resilience (χ^2^ = 0.089). Significant differences were also found in the marital status of these three groups. The high-resilience group had a greater proportion of married people (48.7%) than did the moderate-resilience (23.5%) and low-resilience groups (27.7%) (χ^2^ = 0.023). The results are shown in Table 2.

#### 3.1.2. Occupational Characteristics and Working Environment

Compared with the moderate- (12.9 ± 7.9 years) and low-resilient (14.4 ± 9.7 years) participants, the highly resilient participants had more work experience (18.6 ± 10.9 years) (*p* < 0.001). The data are shown in Table 2. The number of weekly assignments was similar across all groups, with no significant differences (*p* = 0.704). The average weekly working hours also showed no significant differences between the groups (*p* = 0.194). The distribution of professional qualifications significantly differed. In the group with high resilience, 39.7% had the RettSan qualification, whereas 19.0% of the group with moderate resilience and 41.4% of the group with low resilience had the RettSan qualification (pχ^2^ = 0.019). No significant differences were found between the resilience groups with respect to the working environment (metropolis, large city, small town, rural) (pχ^2^ = 0.795). Neither did the distribution of shift working hours differ significantly between the groups (pχ^2^ = 0.407). The participants in managerial positions were more frequently represented in the highly resilient (40.4%) than in the moderately resilient (17.3%) and the low-resilience (42.2%) groups (pχ^2^ = 0.014).

### 3.2. Results of the Resilient Groups in Relation to the Irritation Scale (IS) and Burnout (MBI)

The results of the irritation scale (IS) and the Maslach Burnout Inventory (MBI) are presented below in the context of the various resilience groups in German emergency services. These results revealed significant differences in irritation and burnout symptoms among the high-, moderate- and low-resilience groups (see Table 3).

The average resilience score was greater in the high-resilience (80.0 ± 5.2) than in the moderate-resilience (69.2 ± 1.7), and low-resilience (57.8 ± 8.2) groups. As expected, the differences were significant (*p* < 0.001).

The participants with high resilience had lower scores for cognitive irritation (4.9 ± 2.3) than those with moderate (5.7 ± 1.8) and low resilience (6.1 ± 1.8) (*p* < 0.001). Similarly, the emotional irritation scores were significantly lower in the high-resilience (4.7 ± 1.9) than in the moderate- (5.7 ± 1.5) and low-resilience groups (6.3 ± 1.7) (*p* < 0.001). The total irritation score was also lower in the high-resilience group (4.6 ± 1.9) than in the moderate- (5.7 ± 1.6) and low-resilience (6.4 ± 1.8) groups (*p* < 0.001).

The group with high resilience had lower scores for emotional exhaustion (2.1 ± 1.4) than did the groups with moderate (3.0 ± 1.6) and low resilience (3.3 ± 1.5) (*p* < 0.001). The average scores of the subjects with low resilience corresponded to high expression of the emotional exhaustion dimension (≥3.20 points). The participants in the high-resilience group had lower scores for cynicism (1.7 ± 1.5) than those in the moderate- (2.4 ± 1.5) and low-resilience (2.5 ± 1.6) groups (*p* < 0.001). The average values of the subjects with moderate and low resilience corresponded to a high level of the cynicism dimension (≥2.20 points). Personnel accomplishment was highest in the high-resilience group (5.3 ± 0.7), corresponding to a high level of the dimension (≥5.00 points), whereas it was lower in the moderate-resilience (4.7 ± 0.8) and low-resilience groups (4.4 ± 1.0) (*p* < 0.001), in the range of the average level (4.01–4.99 points). The MBI total score, which is a measure of overall burnout risk, was lower in the high-resilience group (1.9 ± 1.0) than in the moderate-resilience (2.6 ± 1.1) and low-resilience (2.9 ± 1.1) groups (*p* < 0.001). These two resilience groups are classified according to their severity in the category “some burnout symptoms”.

In summary, higher resilience is associated with lower scores on the irritation scale, lower levels of the burnout dimensions, and no burnout risk.

### 3.3. Correlation Results: Spearman-Rho Analysis of the Resilience Score, Irritation Scales, Burnout Dimensions, and Job-Related Data

Spearman correlation analysis (Table 4) revealed several significant correlations between age, professional experience (PE), number of assignments (alerts), weekly working hours (hours), emotional exhaustion (EE), cynicism (CY), personnel accomplishment (PA), cognitive irritation (Cog), emotional irritation (Emo), total irritation score (Total), and resilience score (RS).

#### 3.3.1. Occupational Data

Age was strongly positively correlated with work experience (ρ = 0.891, *p* < 0.001). There were further weak positive correlations between age and hours worked per week (ρ = 0.206, *p* < 0.001), performance on the MBI (ρ = 0.207, *p* < 0.001), and the resilience score (ρ = 0.154, *p* = 0.009). As expected, work experience was strongly positively correlated with age (ρ = 0.891, *p* < 0.001). There were also weak positive correlations between work experience and the number of hours worked per week (ρ = 0.203, *p* < 0.001), performance on the MBI (ρ = 0.215, *p* = 0.008), and the resilience score (ρ = 0.157, *p* = 0.008). The number of assignments had a weak positive correlation with weekly working hours (ρ = 0.184, *p* = 0.002). Weekly working hours always had a weak positive correlation with age (ρ = 0.206, *p* < 0.001) and work experience (ρ = 0.203, *p* < 0.001), among other factors. Weekly working hours were also positively correlated with the MBI dimensions of emotional exhaustion (ρ = 0.184, *p* = 0.002) and cynicism (ρ = 0.145, *p* = 0.015) as well as the scales of irritation: cognitive irritation (ρ = 0.145, *p* = 0.015), emotional irritation (ρ = 0.146, *p* = 0.014), total irritation score (ρ = 0.202, *p* < 0.001), and RS (ρ = 0.212, *p* < 0.001).

#### 3.3.2. Correlations between the Stress Parameter Scale and Burnout Dimension

As expected, emotional exhaustion on the MBI showed a strong positive correlation with cynicism (ρ = 0.702, *p* < 0.001) and a strong negative correlation with MBI performance (ρ = −0.251, *p* < 0.001) (see Table 4). Emotional exhaustion was also strongly positively correlated with cognitive irritation (ρ = 0.531, *p* < 0.001) and emotional irritation (ρ = 0.514, *p* < 0.001), as did the total irritation score (r = 0.910, *p* < 0.001). A further negative correlation was found between the resilience score (RS) (ρ = −0.405, *p* < 0.001) and emotional exhaustion. The cynicism dimension presented a strong positive correlation with the total irritation score (ρ = 0.870, *p* < 0.001) and moderate positive correlations with both cognitive (ρ = 0.368, *p* < 0.001) and emotional irritation (ρ = 0.448, *p* < 0.001). Cynicism was moderately negatively correlated with the resilience score (ρ = −0.306, *p* < 0.001). Performance was weakly negatively correlated with cognitive irritation (ρ = −0.112, *p* = 0.060), emotional irritation (ρ = −0.259, *p* < 0.001), and the total irritation score (ρ = −0.188, *p* = 0.001), and moderately positively correlated with the resilience score (ρ = 0.499, *p* < 0.001).

As expected, cognitive irritation was strongly correlated with the total irritation score (ρ = 0.802, *p* < 0.001) and emotional irritation score (ρ = 0.507, *p* < 0.001). Emotional irritation was strongly correlated with the total irritation score (ρ = 0.870, *p* < 0.001). Both cognitive and emotional irritation were negatively correlated with the RS score (ρ = −0.302. *p* < 0.001 and ρ = −0.461. *p* < 0.001, respectively).

In summary, the analysis revealed complex relationships between age, professional experience, work-related factors, burnout dimensions, irritation, and resilience. Higher resilience scores are associated with lower scores for emotional exhaustion, cynicism, and irritation while lower scores are associated with the performance dimension (which is good).

### 3.4. General Linear Model (GLM) Analysis of Sociodemographic and Occupational Data Related to Emergency Services

The results of a general linear model (GLM) analysis are presented below.

#### 3.4.1. Results of the GLM Analysis of Occupation-Related Data

The results are summarized in Table 5 below. The constant terms were age, job retention, and resilience rating, which significantly influenced the dependent variables in the corrected model. This model could explain at least 8% of the variance (cognitive irritation and cynicism) up to a maximum of 21% (performance).

#### 3.4.2. Further Model in the GLM Analysis

In the model presented below, the following variables were considered as dependent variables: age, work experience, marital status, children, qualifications, managerial function, and resilience rating. Together, these variables explained 10–23% of the variance in the results, but no further effects were found when considered alone. The results are not presented in tabular form.

In summary, resilience is a strong predictor of cognitive and emotional irritation, as well as all measured dimensions of burnout. Higher resilience scores are associated with less irritation and fewer symptoms of burnout. Age and work experience had no (relevant) significant influence on the variables examined in this analysis.

Resilience had a significantly moderate effect on cognitive irritation (*p* < 0.001, η^2^ = 0.133), whereas work experience had a significantly mild effect, and age had no effect. Furthermore, resilience had large effects on the total irritation score (*p* < 0.001, η^2^ = 0.184), emotional exhaustion (*p* < 0.001, η^2^ = 0.165), performance (*p* < 0.001, η^2^ = 0.142), and the Kalimo et al. burnout rating (*p* < 0.001, η^2^ = 0.154).

## 4. Discussion

This study investigated resilience as a characteristic of a safety culture in German emergency services and its effects on mental stress in the form of irritation and burnout. More than one-third of rescue service personnel have a low level of resilience, whereas a further 20% have a moderate, expandable level of resilience. The most striking results are as follows:

(1) Compared with the moderate- and low-resilience groups, the high-resilience group had lower scores for emotional exhaustion, cynicism, and better job performance.
(2) Significant sociodemographic and occupational factors that significantly differed between the resilient groups were age, marital status, partnership, children, work experience, qualifications, and managerial function.
(3) However, the sociodemographic and work-related factors correlated only weakly at best with performance on the MBI, the resilience score, or the irritation scales. Higher resilience scores correlated moderately with lower scores for cognitive and emotional irritation and lower burnout symptoms.
(4) No relevant effects were detected in a test of intersubject effects. Only age, job retention, and resilience rating could be explained as constant terms between 8% and 21% of the variance in cognitive irritation, cynicism, and performance. Individual effects were lost.

The results clearly support H1: “A higher resilience level is associated with lower stress, manifested in reduced irritation and lower burnout symptoms”. The study revealed that rescue workers with higher resilience scores had significantly lower scores for cognitive and emotional irritation. Cognitive irritation, characterized by constant rumination about work problems, and emotional irritation, manifested by increased irritability and aggression, are significantly lower in highly resilient individuals. These results are consistent with those of previous studies [11], suggesting that resilience acts as a buffer against stressors and promotes mental recovery. Thus, the advanced research question cannot be confirmed: Sociodemographic factors (e.g., age, children, marital status) and job-related factors (e.g., work experience, location of assignments) have no significant influence on the results.

Participants with a higher level of resilience showed less emotional exhaustion and cynicism and had a higher level of professional performance. Cynicism, which reflects a distant and negative attitude toward work and patients, was also less pronounced in paramedics with lower resilience. The performance was lowest in the group with low resilience. The risk of burnout was similar. The lower the resilience group is, the greater the risk of burnout. The correlation and GLM analyses confirmed that resilience has a protective effect against burnout symptoms and strengthens the ability of rescue workers to cope with high professional demands.

### 4.1. Discussion of the Results with National and International Literature

In the following, the study results are discussed in the context of the international literature on resilience and burnout in healthcare and emergency services. There are no studies on the irritation of emergency service personnel at the international research level. German veterinarians, for example, had even higher scores for the total cognitive, emotional irritation, and irritation indices than did those in resilience Group I (low resilience) of the German emergency services described here, who had the next highest scores on the irritation scales [53].

A study of healthcare workers in Portugal examined the role of resilience in reducing burnout among healthcare workers during the COVID-19 pandemic [54]. The results revealed that greater resilience was associated with lower scores for emotional exhaustion and depersonalization and higher scores for personal achievement. Another international study assessed burnout and resilience among academic health professionals [55]. The results revealed a significant negative correlation between resilience and burnout. Higher resilience scores were associated with lower burnout scores. A cross-sectional study examined the relationship between psychological resilience and occupational quality of life in psychiatric nurses in Saudi Arabia [56]. The results revealed that greater resilience was correlated with better occupational quality of life and lower rates of burnout. Resilient nurses were better able to cope with the challenges of the profession and experienced fewer symptoms of burnout. Another study examined resilience, burnout, work engagement, and intention to quit among nurses during the COVID-19 pandemic, with the results confirming that greater resilience was associated with lower rates of burnout and, in addition, with a lower intention to leave the profession. This study emphasized the importance of resilience as a protective factor against the negative effects of occupational stress [57].

A qualitative study with eight paramedics revealed that common emotions after difficult missions were feelings of inadequacy, low self-esteem, and “not being good enough” [58]. These emotions were difficult to overcome and were often linked to deep-rooted shame for those involved. The capacity for recovery and resilience varied but showed a positive trend in cultures that encouraged sharing with colleagues and supported personal self-reflection on the causes of the critical event [58]. However, reduced empathy also serves as an emotional shield during missions, and actively dealing with one’s emotions appears to protect against stress [23].

In contrast to the findings of previous studies, sociodemographic or job-related factors had no relevant influence on stress in these three resilience groups in the present study. One study examined the effects of resilience on burnout in nurses during the COVID-19 pandemic and reported significant associations between sociodemographic and job-related variables, such as sex, type of hospital, department, and duration [59]. A study from Spain examined 325 healthcare workers and reported significant correlations between sociodemographic variables and burnout. Age and gender play important roles, with younger age groups and women showing higher rates of burnout. Resilience has been identified as a moderating factor that mitigates the negative effects of burnout [60]. The results of a study among 117 U. S. workers showed that hardiness is a protective factor against burnout in both men and women and that older employees are less susceptible to burnout [38]. A study from Singapore showed that sociodemographic variables such as age, sex, and ethnicity significantly influenced resilience and burnout scores. Younger healthcare professionals and those with less social support presented higher rates of burnout [61]. A study from Malaysia examined 394 healthcare workers and reported that longer working hours were significantly associated with higher levels of burnout and lower resilience. A higher income, on the other hand, was associated with greater resilience and better quality of life. A reduction in working hours could improve resilience and reduce burnout [62]. Another study of 130 nurses in urology clinics in Poland reported that both job satisfaction and burnout levels were strongly influenced by sociodemographic factors such as age, sex, and work experience. Younger nurses and those with less professional experience had higher burnout rates [63].

Previous studies have shown that resilience has a decisive influence on the development of burnout and the improvement of professional quality of life in the healthcare sector. International studies have consistently shown that higher resilience scores are correlated with lower burnout rates, better job satisfaction, and fewer intentions to quit. Moreover, resilience clearly acts as a protective factor against the negative effects of occupational stress and difficult assignments. Given these findings, the following chapter examines the importance of strengthening resilience as a targeted health promotion and prevention measure. It explains how specific strategies and programs to promote resilience can be implemented in the healthcare sector to sustainably improve employees’ mental health and well-being.

### 4.2. The Importance of Strengthening Resilience as a Health Promotion and Prevention Measure and Safety Culture in Companies

The data from resilience studies are inconsistent. Studies have shown the effects of interventions based on strengthening resilience and the health and well-being of emergency service personnel [64]. Notably, when such programs are implemented, resilience training should be offered sustainably or not interrupted [64]. A meta-analysis revealed that interventions based on mindfulness and cognitive behavioral therapy are effective in reducing stress, anxiety, and depression [65]. Inconsistent results were found in a meta-analysis of healthcare trainees (including paramedics). There is very little evidence of the effect of resilience training on resilience, anxiety, and stress or stress perception after the intervention. The heterogeneity of the interventions was partly due to the lack of short-, medium- or long-term data for comparison [66]. Another meta-analysis of military personnel found no evidence that different conceptualizations of psychological resilience in various research designs have strong predictive power for the mental health and functioning of military personnel [67].

These inconsistent results indicate that further research is needed. How resilience interventions are designed and implemented varies greatly. There are many reasons for this. Differences in methods, content, and approaches (e.g., mindfulness training, cognitive behavioral therapy, and physical activities) contribute to the varying results. Some studies use short, intensive programs, whereas others use long-term, less intensive approaches. Studies on resilience interventions are aimed at different target groups, from healthcare providers and military personnel to schoolchildren and students. The specific needs and starting conditions of these groups vary considerably, which can lead to different results. Various scales and questionnaires are used to measure resilience, stress, and burnout, which differ in their validity and reliability. The use of different measurement instruments can contribute to inconsistent results. Differences in study quality and design, such as cross-sectional versus longitudinal studies, sample size, control groups, and randomization, influence the results. Methodological weaknesses and biases can affect the comparability and reliability of studies. Resilience training is certainly a behavioral approach that does not always appear to be successful in group sessions as individual aspects may not be taken into account. A meta-analysis revealed that 70% of training sessions take place in groups [68].

Research appears to be particularly important in the workplace setting as the world of work is the largest prevention setting. Here, continuous and structured resilience measures can strengthen individual resilience and contribute to general health promotion and prevention. Given the daily challenges and stressors that emergency service personnel are exposed to, targeted resilience training in the professional environment can be particularly effective, especially in strengthening team cohesion.

Public safety organizations emphasize stress management and resilience to improve mental health in the workplace. The most common approach they used was multisession training. Few organizations have targeted their interventions directly at the employee level to change the work environment or way of working [68].

In this study, the concepts of the VUCA and BANI worlds were introduced to illustrate the complex and dynamic conditions under which EMS personnel operate. These concepts extend beyond traditional notions of stressors in emergency medical services and encompass the uncertainties, brittleness, and nonlinear challenges that EMS personnel face daily. While the study primarily focused on resilience as a key factor in managing stress and burnout, the VUCA and BANI models provide a conceptual framework that highlights why resilience strategies are particularly relevant for EMS personnel. They underscore that working in emergency services is demanding not only because of direct patient care but also due to the complex and unpredictable environments in which these operations take place. Understanding these additional stressors can lead to more targeted measures to enhance the resilience and well-being of EMS workers. The findings of this study have several important implications for practice and future research in the field of emergency medical services. First, the results highlight the central role of resilience in managing stress, irritation, and burnout among EMS personnel. This underscores the need to develop and implement targeted interventions to promote resilience within emergency services. Such interventions could include regular resilience training, fostering team cohesion and social support, and creating a supportive work environment.

Furthermore, the study shows that higher levels of resilience are associated with lower levels of emotional exhaustion and cynicism, as well as better job performance. These findings and findings from the literature suggest that enhancing resilience can not only promote the mental health and well-being of EMS personnel but also improve their performance and job satisfaction.

Strengthening resilience is a dynamic and continuous process in which various areas of competence are systematically trained to increase individual and collective resilience [69]. According to Aman and Egger, typical areas of expertise are (1) improvisation and willingness to learn; (2) optimism, positive self-assessment, and assessment of others; (3) acceptance and connection to reality; (4) solution orientation and creativity; (5) self-regulation and self-care; (6) personal responsibility and creative power; (7) relationships, appreciation, and cooperation; and (8) shaping the future and developing a vision [69].

As part of resilience training, various levels can be addressed to achieve holistic promotion of resilience. This can be achieved

(1) At the mindset level (e.g., mindset that strengthens resilience, mindfulness);
(2) At the embodiment level by developing better body awareness and creating a sense of well-being;
(3) At the level of interaction with others (e.g., promoting social engagement, building security);
(4) Through the context design, such as relationship prevention, with, for example, creating a resilience-friendly environment [64]. Depending on the method, different approaches can be used, making studies on strengthening resilience difficult to compare.

The inconsistent data situation and the large number of different intervention strategies illustrate the complexity of this topic. Nevertheless, the authors argued that systematic and sustainable implementation of resilience measures is necessary to promote mental health and well-being.

In addition to individual resilience, the safety culture of companies is crucial. A strong safety culture not only contributes to the prevention of physical accidents but also promotes the mental well-being of employees. In emergency services, in particular, where employees are exposed to extreme stress and dangerous situations daily, a safety culture promoting resilience plays a key role. One review concludes that huddles (short meetings between team members to briefly exchange information) have a largely positive impact on teamwork and job satisfaction [70]. Another review examined building a foundation of trust and respect through simulation, training, and mindful communication and the effects on improved patient safety and teamwork/communication [71].

Integrating resilience measures into a company’s safety culture can strengthen team cohesion and increase overall resilience. To be effective, these measures must be tailored to the specific requirements and challenges of the emergency services organization. There is no one-size-fits-all recipe.

### 4.3. Strengths and Weaknesses of the Present Work

A particular strength of this study is the investigation of irritation using the irritation scale as an occupational health prevention tool. This tool has been little researched to date. By showing that greater resilience is associated with lower levels of cognitive and emotional irritation, this study provides valuable insights into how irritation can be used as an indicator for early prevention measures in companies.

With a total of 85,000 rescue service employees in Germany (year 2021) [72], the sample of 280 people represents only a very small percentage (approx. 0.33%). This may limit generalizability as specific regional or organizational differences may not be sufficiently represented. Nevertheless, the number of 280 participants provides a solid basis for identifying trends and correlations within the occupational group.

Recruiting participants via various channels (social media, email distribution lists, specialist journals) ensures a diverse and representative sample. Recruitment via personal contacts and networks could lead to bias in that people with a higher affinity for the topic or better mental health are overrepresented. This could bias the results in a positive direction and not accurately reflect the actual stress and resilience levels of the overall population. The study focuses on full-time emergency service employees and excludes volunteers. Although volunteers comprise a significant proportion of emergency services, their specific stress and resilience levels were not considered. Not all possible factors influencing resilience and mental stress were taken into account. Factors such as genetic predisposition, previous traumatic experiences or individual coping strategies, self-perception, self-control, self-efficacy, and social skills could also play a role and were not examined in this study. Similarly, current stress levels in the rescue service were not surveyed. The study assumes known physical and psychological stresses although there could be different regional stresses due to the decentralized organization of the rescue service, for example. This also includes the organizational structure of the rescue service (e.g., professional fire departments, aid organizations), which can have a considerable influence on the results [73]. One limitation of this study is the lack of a detailed examination of the various factors that may contribute to burnout and irritability among participants. Factors such as individual stress levels, organizational conditions, and work relationships were not directly assessed. This omission means that while we can identify correlations between resilience and outcomes like burnout and irritability, we cannot conclusively determine the underlying causes of these states.

## 5. Conclusions

This study clearly revealed that resilience, as a component of the safety culture in emergency services, plays a decisive role in the extent of stress in the form of irritation and burnout. However, more than one-third of the participating emergency service personnel exhibited a low level of resilience, and an additional 20% exhibited only a moderate level, underscoring the urgency of measures to promote resilience.

As sociodemographic and job-related factors have no relevant influence on resilience and stress, individual resilience itself appears to be the decisive factor in reducing irritation and burnout. This finding suggests that organizations should develop and implement targeted measures to strengthen the resilience of rescue workers.

The practical implications of this study include the need for regular education and training programs to strengthen resilience, establish a supportive work environment, and promote team cohesion and social support within emergency services. These measures not only improve the mental health of rescue workers but also increase their professional performance and satisfaction. Overall, the study shows that resilience is a key factor for health promotion and prevention in emergency services. Future research should aim to develop specific intervention strategies and evaluate their effectiveness in different rescue service contexts. This is the only way to safeguard rescue workers’ mental and physical health in the long term and improve the quality of emergency care.

## Figures and Tables

**Table 1:** Degree of the MBI dimensions.

Burnout Dimension	Degree of Burnout (Points)		
	Low	Average	High
Emotional exhaustion	≤2.00	2.01–3.19	≥3.20
Cynicism	≤1.00	1.01–2.19	≥2.20
Personnel accomplishment	≤4.00	4.01–4.99	≥5.00

**Table 2:** Comparison of resilience groups in the German emergency services: demographic characteristics, professional experience, and working conditions.

		Resilience Groups				
		High n = 117	Moderate n = 58	Low n = 110	p_KW_ or χ^2^	p_Bon_
Age (years)	MW ± SD Median (Min.Max) 95%CI	40.4 ± 10.5 40 (18–61) [38.5–42.4]	34.4 ± 8.6 33 (22–62) [32.1–36.7]	36.5 ± 10.5 34 (19–62) [34.5–38.5]	**<0.001**	High/Moderate (0.001) High/Low (0.012)
Sex (n. (%))	Male (n =207)	92 (44.4)	44 (21.3)	71 (34.3)	0.089	-
	Female (n = 76)	25 (32.9)	13 (17.1)	38 (50)		
	Not specified (n = 2)	0	1 (50)	1 (50)		
Family status (n. (%))	Single (n = 141)	48 (34)	28 (19.9)	64 (46.1)	**0.023**	-
	Married (n = 119)	58 (48.7)	28 (23.5)	33 (27.7)		
	Widowed (n = 1)	1 (100)	0	0		
	Divorced (n = 24)	10 (41.7)	2 (8.3)	12 (50)		
Lives in partner-ship (n. (%))	Yes (n = 216)	94 (43.5)	46 (21.3)	76 (35.2)	**0.042**	-
	No (n = 64)	19 (29.7)	12 (18.8)	33 (41.6)		
	Not specified (n = 5)	5 (80)	0	1 (20)		
Children (n. (%))	Yes (n = 23) No (n = 260) Not specified (n = 2)	65 (50)	29 (22.3)	36 (27.7)	**0.004**	-
		51 (33.3)	28 (18.3)	74 (48.4)		
		1 (50)	1 (50)	0		
Caring for relatives (n. (%))		9 (39.1)	3 (13)	11 (47.8)	0.303	-
		106 (40.8)	55 (21.2)	99 (38.1)		
		2 (100)	0	0		
Professional experience (years)	MW ± SD Median (Min.Max) 95%CI	18.6 ± 10.9 17 (1–42) [16.6–20.6]	12.9 ± 7.9 10 (1–42) [10.8–15]	14.4 ± 9.7 12 (1–42) [12.5–16.2]	**<0.001**	High/Moderate (0.004) High/Low (0.007)
Number of missions		6.4 ± 3.2 6 (0–24) [5.8–7.0]	6.4 ± 2.6 6 (0–13) [5.7–7.19	6.5 ± 2.4 6 (2–14) [6.1–6.4]	0.704	-
Weekly working hours		45.4 ± 11.4 48 (6–80) [43.3–47.5]	45.8 ± 11.5 48 (4–65) [42.7–48.8]	47.4 ± 10.0 48 (5–72) [45.5–49.3]	0.194	-
Qualification (n. (%))	RettSan (n = 58)	23 (39.7)	11 (19.0)	24 (41.4)	**0.019**	-
	RettAss (n = 15)	8 (53.3)	1 (6.7)	6 (40)		
	NotSan (n = 193)	71 (36.8)	45 (23.3)	77 (39.9)		
	EP (n = 13)	11 (84.6)	1 (7.7)	1 (7.7)		
	Not specified (n = 6)	4 (66.7)	0	2 (33.3)		
Working environment (n. (%))	Metropolis (n = 22)	11 (50)	3 (13.6)	8 (36.4)	0.795	-
	Large city (n = 78)	28 (35.9)	19 (24.4)	31 (39.7)		
	Small town (n = 107)	42 (39.3)	22 (20.6)	43 (40.2)		
	Rural (n = 78)	36 (n = 46.2)	14 (17.9)	28 (35.9)		
Shift work (n. (%))	8 h (n = 41)	22 (53.7)	6 (14.6)	13 (31.7)	0.407	-
	12 h (n = 147)	54 (36.7)	33 (22.4)	60 (40.8)		
	24 h (n = 97)	41 (42.3)	19 (19.6)	37 (38.1)		
Leading function (n. (%))	Yes	91 (40.4)	39 (17.3)	95 (42.2)	**0.014**	**-**
	No	26 (43.3)	19 (31.7)	15 (25)		

Notes. _KW_ = Kruskal–Wallis test, Bon = pairwise comparison with Bonferroni correction, χ^2^ = Chi-square. RettSan = paramedic, RettAss = paramedic, NotSan = emergency paramedic, EP = emergency physician. The significant differences are in bold.

**Table 3:** Results of the mental stress according to resource groups.

	Resilience Groups				
	III (High) n = 117	II (Moderate) n = 578	I (Low) n = 110	p_KW_	p_Bon_
	MW ± SD Median (Min.Max) 95%CI				
RS-13					
Resilience score	80.0 ± 5.2 79 (73–91) [79.09–81.00]	69.2 ± 1.7 69 (67–72) [68.75–69.67]	57.8 ± 8.2 60 (21–66) [56.20–5.32]	**<0.001**	III/II, III/I, II/I < 0.001
IS					
Cognitive	4.9 ± 2.3 5 (1–9) [4.46–5.29]	5.68 ± 1.82 6 (1–9) [5.20–6.17]	6.1 ± 1.8 6 (2–9) [5.78–6.48]	**<0.001**	III/II = 0.049; III/I < 0.001
Emotional	4.7 ± 1.9 5 (1–9) [4.35–5.04]	5.7 ± 1.5 6 (3–9) [5.32–6.12]	6.3 ± 1.7 6 (1–9) [5.97–6.62]	**<0.001**	III/II = 0.002; III/I < 0.001
Total	4.6 ± 1.9 5 (1–9) [4.34–5.05]	5.7 ± 1.6 6 (3–9) [5.25–6.11]	6.4 ± 1.8 6 (2–9) [6.06–6.79]	**<0.001**	III/II = 0.005; III/I < 0.001
MBI-GS					
Emotional exhaustion	2.1 ± 1.4 1.6 (0–6) [1.81–2.35]	3.0 ± 1.6 2.6 (0–6) [2.53–3.38]	3.3 ± 1.5 3 (0–6) [2.96–3.55]	**<0.001**	III/II = 0.001; III/I < 0.001
Cynicism	1.7 ± 1.5 1.2 (0–6) [1.42–1.98]	2.4 ± 1.5 2 (0–6) [2.00–2.81]	2.5 ± 1.6 2.4 (0–6) [2.24–2.85]	**<0.001**	III/II = 0.004; III/I < 0.001
Personnel accomplishment	5.3 ± 0.7 5.5 (3–6) [5.18–5.46]	4.7 ± 0.8 4.8 (3–6) [4.51–4.92]	4.4 ± 1.0 4.5 (1–6) [4.16–4.55]	**<0.001**	III/II, III/I < 0.001
Total score	1.9 ± 1.0 1.7 (0–5) [1.73–2.11]	2.6 ± 1.1 2.1 (1–5) [2.32–2.88]	2.9 ± 1.1 2.7 (1–6) [2.66–3.08]	**<0.001**	III/II, III/I < 0.001

Notes. KW = Kruskal–Wallis test, Bon = pairwise comparison with Bonferroni correction. The significant differences are in bold.

**Table 4:** Results from the Spearman rho analysis of resilience scores, irritation scales, burnout dimensions and job-related data.

	Age	PE	Alerts	Hours	EE	CY	PA	Kalimo	Cog	Emo	Total	RS
Age		0.891 ***		0.206 ***			0.207 ***					0.154 **
PE	0.891 ***			0.203 ***			0.215 **					0.157 **
Alerts				0.184 **								
Hours	0.206 ***	0.203 ***	0.191 **		0.184 **	0.145 *		0.184 **	0.146 *	0.202 ***	0.212 ***	
EE						0.702 ***	−0.251 ***	0.910 ***	0.531 ***	0.514 ***	0.589 ***	−0.405 ***
CY							−0.273 ***	0.870 ***	0.368 ***	0.448 ***	0.463 ***	−0.306 ***
PA								−0.458 ***	−0.112 **	−0.259 ***	−0.188 **	0.499 ***
Cog										0.507 ***	0.802 ***	−0.302 ***
Emo											0.870 ***	−0.461 ***
Total												−0.459 ***

Notes. PE = professional experience, Alerts = number of missions, Hours = weekly working hours, EE = MBI Emotional exhaustion, CY = MBI cynicism, PA = MBI personal accomplishment, Kog = IS cognitive, Emo = IS emotional, Total = IS total score, RS = resilience score. Interpretation of ρ according to Cohen (1988): weak correlation ρ = 0.10, moderate correlation ρ = 0.30, strong correlation ρ = 0.50. The respective color intensity shows the strength of the correlation. Green = positive and red = negative correlations. * *p* < 0.05, ** *p* < 0.01, *** *p* < 0.001.

**Table 5:** Results of the general linear model (GLM) analysis: influences of age, professional experience, and resilience on irritation and burnout in the emergency services.

	GLM (Test of Inter-Subject Effects) with Bootstrap													
	Resilience Groups													
Dependent Variables	III (High) n = 115	II (Moderate) n = 57	I (Low) n = 108	Corrected Model					Age		Work Experience		Resilience Rating	
	Estimated Marginal Mean MW ± Std. Error [95% CI]			R^2^	Corr. R^2^	F	p	η^2^	p	η^2^	p	η^2^	p	η^2^
IS cognitive	4.808 ± 0.190 [4.435–5.182]	5.694 ± 0.268 [5.167–6.222]	6.119 ± 0.193 [5.739–6.500]	0.083	0.070	6.358	**<0.001**	0.083	0.192	0.006	**0.031**	0.017	**<0.001**	0.133
IS emotional	4.718 ± 0.163 [4.397–5.038]	5.741 ± 0.230 [5.287–6.194]	6.301 ± 0.166 [5.974–6.628]	0.153	0.146	13.142	**<0.001**	0.158	0.516	0.002	0.109	0.009	**<0.001**	0.077
IS total	4.678 ± 0.172 [4.339–5.016]	5.716 ± 0.243 [5.238–6.194]	6.438 ± 0.175 [6.093–6.783]	0.160	0.148	13.317	**<0.001**	0.160	0.416	0.002	0.941	0.001	**<0.001**	0.184
MBI emotional exhaustion	2.008 ± 0.141 [1.730–2.286]	3.051 ± 0.200 [2.658–3.444]	3.291 ± [0.144] [3.008–3.575]	0.140	0.128	11.426	**<0.001**	0.140	0.207	0.006	**0.037**	0.015	**<0.001**	0.165
MBI cynicism	1.639 ± 0.144 [1.355–1.923]	2.466 ± 0.204 [2.065–2.868]	2.592 ± 0.147 [2.303–2.882]	0.084	0.070	6.381	**<0.001**	0.084	0.203	0.006	0.134	0.008	**<0.001**	0.078
MBI personal accomplishment	5.288 ± 0.081 [5.127–5.448]	4.773 ± 0.115 [4.546–4.999]	4.356 ± 0.083 [4.192–4.519]	0.213	0.202	18.977	**<0.001**	0.158	0.449	0.002	0.263	0.004	**<0.001**	0.142
MBI Kalimo	1.879 ± 0.098 [1.687–2.071]	2.646 ± 0.138 [2.734–2.918	2.900 ± 0.100 [2.704–3.096]	0.171	0.159	14.401	**<0.001**	0.160	0.637	0.001	0.676	0.001	**<0.001**	0.154

Notes. η^2^ < 0.06 (mild effect), η^2^ = 0.06 bis 0.14 (moderate effect), η^2^ > 0.14 (high effect). The respective color intensity shows the strength of the effects. The significant differences are in bold.

## Data Availability

There are no plans to grant access to the full protocol, participant-level datasets, or statistical codes as data contain potentially identifying information.
